# Can Post-Error Dynamics Explain Sequential Reaction Time Patterns?

**DOI:** 10.3389/fpsyg.2012.00213

**Published:** 2012-07-16

**Authors:** Stephanie Goldfarb, KongFatt Wong-Lin, Michael Schwemmer, Naomi Ehrich Leonard, Philip Holmes

**Affiliations:** ^1^Princeton Neuroscience Institute, Princeton UniversityNJ, USA; ^2^Department of Mechanical and Aerospace Engineering, Princeton UniversityNJ, USA; ^3^Intelligent Systems Research Centre, University of Ulster, Magee CampusNorthern Ireland, UK; ^4^Program in Applied and Computational Mathematics, Princeton UniversityNJ, USA

**Keywords:** drift diffusion model, error rate, perceptual decision making, post-error slowing, reaction time, sequential effects

## Abstract

We investigate human error dynamics in sequential two-alternative choice tasks. When subjects repeatedly discriminate between two stimuli, their error rates and reaction times (RTs) systematically depend on prior sequences of stimuli. We analyze these sequential effects on RTs, separating error and correct responses, and identify a sequential RT tradeoff: a sequence of stimuli which yields a relatively fast RT on error trials will produce a relatively slow RT on correct trials and vice versa. We reanalyze previous data and acquire and analyze new data in a choice task with stimulus sequences generated by a first-order Markov process having unequal probabilities of repetitions and alternations. We then show that relationships among these stimulus sequences and the corresponding RTs for correct trials, error trials, and averaged over all trials are significantly influenced by the probability of alternations; these relationships have not been captured by previous models. Finally, we show that simple, sequential updates to the initial condition and thresholds of a pure drift diffusion model can account for the trends in RT for correct and error trials. Our results suggest that error-based parameter adjustments are critical to modeling sequential effects.

## 1 Introduction

Efforts to model and predict human behavior are informed by an understanding of the dynamics of error rates (ERs) and reaction times (RTs) in simple tasks. In particular, in two-alternative forced-choice (TAFC) tasks (e.g., Laming, 1968; Link, 1975; Link and Heath, 1975; Ratcliff and Rouder, 1998) human participants are known to slow down after committing an error, and generally to exhibit RTs and ERs that systematically depend on prior stimulus sequences (Bertelson, 1961; Capaldi, 1966; Laming, 1968; Remington, 1969; Kirby, 1976; Vervaeck and Boer, 1980; Soetens et al., 1984, 1985). However, while much previous work has considered post-error slowing and sequential effects separately, we are not aware of studies that explicitly account for interactions among these effects.

Patterns in RTs for individual trials are well documented in the literature. In particular, relative to their mean RTs on correct trials, subjects are known to respond faster on error trials and more slowly immediately following errors (Rabbitt, 1966; Laming, 1979a,b). On average it has been shown that participants return to their mean RT values within two trials after an error (Rabbitt, 1968b). Various models of TAFC tasks have accounted for this post-error slowing (Ratcliff and Rouder, 1998; Dudschig and Jentzsch, 2009). However, to our knowledge the mean RTs on trials following specific sequences of stimuli have not been studied independently for trials ending in an error, and deliberate post-error adjustments have not been incorporated into models of sequential effects.

Moreover, the characteristic patterns in speed and accuracy following sequences of repetitions and alternations are well documented only for tasks in which the stimuli are equally likely. While overall trends in speed and accuracy have received significant attention (Carpenter and Williams, 1995; Ratcliff and Smith, 2004; Bogacz et al., 2006; Simen et al., 2009), for tasks in which the stimuli are not equally likely, such sequential patterns in mean RT have not been considered.

In a majority of TAFC studies, participants are either rewarded equally for overall participation or they are rewarded for each correct response. However, several studies (e.g., Corrado et al., 2005; Feng et al., 2009) have investigated tasks which reward correct responses to one stimulus more highly than another and have shown that reward contingencies influence choice behavior. When reward probabilities or reward values are unequal, participants are known to select the stimulus corresponding to the most probable or most valuable reward more frequently (Corrado et al., 2005; Feng et al., 2009), and they may do so almost optimally (Gao et al., 2011).

When stimuli are equally probable and correct responses are equally rewarded, several effects are known. For small (<500 ms) response to stimulus intervals (RSIs), the behavior typically illustrates automatic facilitation (AF): mean RTs on the current trial are faster if the previous trial was a repetition, regardless of whether the current trial is a repetition or an alternation. For slow RSIs (≈1000 ms), mean RTs on the current trial after a series of alternations are faster if the current trial is a repetition and slower if the current trial is an alternation (Bertelson, 1961; Laming, 1968; Kirby, 1976). This effect is called strategic expectancy (SE), suggesting a relationship between a participant’s expectations and his or her reaction time. Moreover, a transition occurs from AF to SE as RSI increases (Soetens et al., 1985; Jentzsch and Sommer, 2002) and can be illustrated graphically (Audley, 1973). Prior to the present paper, it was unknown whether such a transition from AF to SE could also occur for a constant RSI with increasing probability of alternations, or, more generally, how sequential effects carry over from equally probable to biased stimuli.

**Figure 1:**
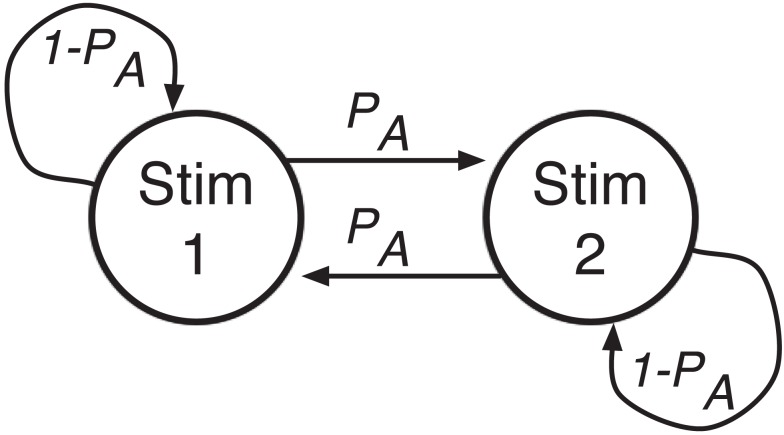
**Stimulus order is generated by a transition-oriented Markov process**. Given current stimulus 1, the next stimulus will be stimulus 2 (an alternation) with probability *P*_A_ and stimulus 1 (a repetition) with probability 1 − *P*_A_.

In this paper, we study sequential patterns in ERs as well as in RTs for error and correct responses independently in TAFC tasks in which stimuli are equally probable or strongly biased toward repetitions or alternations, focusing on sequences of three trials. Stimulus sequences can be biased by specifying stimulus probabilities (state orientation) or by specifying transition probabilities between states (transition orientation), and it is known that these processes produce distinct response patterns in RT (Brodersen et al., 2008). Since we are interested in sequential effects and expectancy, we generate stimuli by a first-order Markov process with unequal (as well as equal) transition probabilities (see Figure 1): The unequal case sets the probability of an alternation (*P*_A_) to be unequal to the probability of a repetition (1 − *P*_A_). Transition probabilities *P*_A_ and 1 − *P*_A_ are held fixed over blocks of trials, and we use relatively long RSIs (800 and 1,000 ms mean), for which SE is most apparent. We reanalyze behavioral data from an equal-probability experiment (Cho et al., 2002), and we collect and analyze a new data set with *P*_A_ set to 10, 50, and 90%. We find significant transition probability effects on RTs for error and correct responses and on ERs.

To further study patterns in RT and ER we extend the pure drift diffusion model (DDM) to account for sequential patterns. The pure DDM describes choice between two alternatives by representing the noisy accumulation of the difference in evidence (logarithmic likelihood) from a given initial condition to one of two decision thresholds. This process is known to mimic aspects of neural integration (Carpenter and Williams, 1995; Gold and Shadlen, 2000; Bogacz et al., 2006; Gold et al., 2008). Adapting the DDM, we propose two simple update mechanisms to vary the initial condition and thresholds from trial to trial, depending on previous stimuli and response correctness. We show how our adapted DDM can account for the observed trends in RT for correct and error trials.

Related TAFC models frequently involve a variant of the leaky competing accumulator (LCA; Usher and McClelland, 2001), featuring two coupled stochastic differential equations which contain multiple parameters to account for leakage (decay of previous evidence) and for the interaction between neural populations. LCA models have been shown to capture sequential effects for equally probable stimuli (Cho et al., 2002; Gao et al., 2009). For certain parameter ranges, it can be shown that the LCA, along with race, inhibition, and other models, reduces to a DDM, and the DDM itself may be extended to account for variability in the model parameters (Ratcliff and Smith, 2004; Bogacz et al., 2006). However, we are aware only of modeling studies that predict both ERs and RTs for sequential effects (Cho et al., 2002; Gao et al., 2009), and these studies did not analyze patterns in error RTs, nor did they incorporate post-error parameter adjustments into the analysis. Bayesian models of TAFC, which can also be represented by DDMs for certain parameter ranges (Liu et al., 2009), have also been used to model sequential effects (Yu and Cohen, 2009; Wilder et al., 2009), but none of these models yet accounts for patterns in errors.

Physiological evidence suggests sources of systematic changes in behavior from trial to trial, providing some neurobiological basis for our proposed update mechanisms. An electroencephalogram (EEG) study has identified a SE pattern in the P300 response (Sommer et al., 1999), an event related potential signal which follows 300–600 ms after unexpected, alternating, stimuli. The prefrontal cortex is also activated following an alternation after frequent repetitions, with greater activation following a longer run of repetitions prior to the alternation (Huettel et al., 2005). In addition, the anterior cingulate cortex (ACC) is known to show increased activity with increased conflict in representation, or alternation of stimuli, and ACC activity has been linked to cognitive control and post-error corrections and corresponding increase in RT (Botvinick et al., 2001). Prior work has incorporated ACC conflict signals into models of sequential and error effects (Jones et al., 2002).

An understanding of the relationship between error correction and sequential biasing mechanisms may allow us to further differentiate between corresponding physiological processes. Such an understanding could have broad implications. Indeed, recent work suggests that the same mechanisms that account for sequential effects also account for sequence learning (Soetens et al., 2004): a general mechanism may therefore lend insight into sequence learning (Soetens et al., 1985; Pashler and Baylis, 1991a,b; Frensch and Miner, 1994), including linguistic processes. Further, better understanding of the mechanisms behind simple discrimination tasks may also allow for improved prediction and prevention of errors.

This paper is organized as follows. In Section 2, we describe two experiments: the first originally reported in Cho et al. (2002) and the second conducted specifically for the present study. We then describe a diffusion model account of participant behavior in the tasks. In Section 3, we describe the experimental results and discuss diffusion model fits to participant behavior. Finally, Section 4 contains further discussion and our conclusions, and it identifies directions for future experimental and modeling work.

## 2 Materials and Methods

In this section, we describe the protocol followed for the two experiments presented in this paper. We then describe a general model of decision making, which accounts for choice behavior with two simple mechanistic adaptations to the pure drift diffusion model (DDM). Finally, we describe a procedure for fitting the model to match participant data in our adapted DDM.

### 2.1 Experiment 1: Error dynamics in unbiased tasks

The first experiment (reanalyzed from Cho et al., 2002) served as a control in which stimulus probabilities were equal and transition probabilities were held constant at 50% throughout the experiment. As the details of the experiment have been described in the literature previously, we discuss them only briefly here. In Experiment 1, six Princeton University undergraduates participated in a task over a single session by identifying the upper or lowercase “o” character on the screen with the appropriate keypress. The index finger was used to identify the uppercase letter, and the middle finger to identify the lowercase letter. Each session consisted of 13 blocks of 120 trials each, and a response to stimulus interval (RSI) of 800 ms was used. Participants received course credit in exchange for their participation in the study. For additional details see Cho et al. (2002). No trials were omitted from our reanalysis.

### 2.2 Experiment 2: Error dynamics in transition-biased tasks

In the second experiment stimulus transition probabilities were varied from block to block, so that in a given block a participant would have a constant high, medium, or low probability of alternations. That is, given the current stimulus 1, a participant would next see the other stimulus 2 with probability *P*_A_ and the same stimulus 1 again with probability 1 − *P*_A_, and the sequence of stimuli would be drawn from a transition-oriented Markov process, as shown in Figure 1.

#### 2.2.1 Participants

Sixteen adults (6 males) participated in exchange for a standard payment of $12 per session of 9 blocks. Participants were recruited from the Princeton University community via announcements posted online and on campus. The experiment was approved by the Institutional Review Panel for Human Subjects of Princeton University, and all participants provided their informed consent prior to participation.

#### 2.2.2 Stimuli

Participants performed an RT version of a motion discrimination task. The visual stimulus consisted of a black screen showing a cloud of white moving dots with a red, stationary fixation dot at its center. The red dot had size 0.30° visual angle, and the white dots had size 0.15° each and moved within a circle of diameter 10° at a speed of 7°/s and a density of 20 dots/degree^2^. On each trial 90% of the white dots would move coherently in a given, “correct” direction, and the remaining white dots would move randomly. The high coherence of motion was selected to ensure that some processing was necessary but that the difficulty of the task would remain low, consistent with other studies of sequential effects. A decision could be indicated with a left or right keypress at any point after dots appeared on the screen. Responses were collected via the standard Macintosh computer keyboard, with the “Z” key used to indicate leftward motion and the “M” key used to indicate rightward motion. The experiment was performed on a Macintosh computer using the Psychophysics Toolbox extension (Brainard, 1997).

#### 2.2.3 Procedure

The participants were instructed to fixate upon the red dot and then determine the direction of the moving dots. They were also instructed to complete the session as quickly and as accurately as possible. Each participant completed 1 session of approximately 60 min duration.

Each session consisted of 9 blocks of 200 trials each in which the *P*_A_ remained fixed at 10, 50, or 90% (3 blocks for each condition). The order of the blocks was constrained to follow a Latin Square design. Participants were allowed a short break between blocks. To minimize anticipatory responding, response to stimulus intervals were drawn from a gamma distribution with a mean of 1 s for each trial, following the convention set in previous sequential RT tasks (Rabbitt, 1966; Simen et al., 2006; Brodersen et al., 2008; Balci et al., 2011). Outlier RTs (less than 100 ms or greater than 900 ms, comprising less than 1.5% of the data) were not included in the analysis. We note that only the outlier was removed from the RT and error analysis; it was included in sorting RR, AR, RA and AA sequences, since it precedes a trial that is included in the analysis. In addition, one participant failed to follow instructions and the corresponding data were omitted from the analysis.

During each block in the session, the subjects received the following feedback. Correct responses were denoted with a short beep sound, and error and premature, anticipatory responses were denoted with a buzz sound. In addition, on every fifth trial, the number of correct responses provided in that block so far was briefly flashed across the screen. This was the only feedback that was provided. Participants were seated at a viewing distance of approximately 60 cm from the screen. Our protocol in Experiment 2 is similar to others in the literature (e.g., Newsome and Pare, 1988; Britten et al., 1992; Ratcliff and McKoon, 2008).

### 2.3 An adapted drift diffusion model

To account for sequential effects and error effects, we consider a simple adaptation of the pure drift diffusion model (DDM) in which the initial condition and thresholds are updated sequentially following each trial (Ratcliff and Rouder, 1998; Ratcliff and Smith, 2004; Bogacz et al., 2006). In the pure DDM, information is accumulated stochastically according to the following equation:

(1) $$dx=\mu dt+\sigma dW,x\left(0\right)=x_{0}.$$

Here *x*(*t*) represents the difference in logarithmic likelihood ratio for the two choices, the drift rate μ (conventionally taken to be positive) represents the difference in incoming evidence for the correct alternative relative to the incorrect alternative, and σ*dW* is a Wiener (white noise) process with mean 0 and variance σ^2^. The evidence thresholds are set at ±*z*, and noisy accumulation continues until *x*(*t*) first crosses either +*z* (a correct decision) or −*z* (an error). If the non-decision time is then given by *T*_nd_ such that RT = DT + *T*_nd_ where DT is the decision time, it can be shown that the mean DT and ER are (Gardiner, 1985; Busemeyer and Townsend, 1992):

(2) $$\langle \text{DT}\rangle =\tilde{z}\tanh(\tilde{z}\tilde{\mu })+\left\{\frac{2\tilde{z}(1-\exp(-2{\tilde{x}}_{0}\tilde{\mu }))}{\exp(2\tilde{z}\tilde{\mu })-\exp(-2\tilde{z}\tilde{\mu })}-{\tilde{x}}_{0}\right\}$$

and

(3) $$\langle \text{ER}\rangle =\frac{1}{1+\exp(2\tilde{z}\tilde{\mu })}-\left\{\frac{1-\exp(-2{\tilde{x}}_{0}\tilde{\mu })}{\exp(2\tilde{z}\tilde{\mu })-\exp(-2\tilde{z}\tilde{\mu })}\right\},$$

in which the parameters have been scaled so that

(4) $$\tilde{z}=\frac{z}{\mu },\tilde{x}=\frac{x_{0}}{\mu },\text{and}\tilde{\mu }={\left(\frac{\mu }{\alpha }\right)}^{2}.$$

Given a non-zero initial condition ${\tilde{x}}_{0},$ mean DTs are different for correct and error trials:

(5) $$\begin{array}{l} \langle {\text{DT}}_{\text{correct}}\rangle =\frac{\exp((\tilde{z}-{\tilde{x}}_{0})\tilde{\mu })}{1-\text{ER}}\\ \times \frac{\left[(\tilde{z}-{\tilde{x}}_{0})\cosh((\tilde{z}+{\tilde{x}}_{0})\tilde{\mu })\sinh(2\tilde{z}\tilde{\mu })-2\tilde{z}\sinh((\tilde{z}-{\tilde{x}}_{0})\tilde{\mu })\right]}{\sinh^{2}(2\tilde{z}\tilde{\mu })} \end{array}$$

(6) $$\begin{array}{l} \langle {\text{DT}}_{\text{error}}\rangle =\frac{\exp(-(\tilde{z}-{\tilde{x}}_{0})\tilde{\mu })}{\text{ER}}\\ \times \frac{\left[(\tilde{z}+{\tilde{x}}_{0})\cosh((\tilde{z}-{\tilde{x}}_{0})\tilde{\mu })\sinh(2\tilde{z}\tilde{\mu })-2\tilde{z}\sinh((\tilde{z}+{\tilde{x}}_{0})\tilde{\mu })\right]}{\sinh^{2}(2\tilde{z}\tilde{\mu })} \end{array}$$

See the Appendix for derivations of equations (5 and 6).

The simplicity and analytical tractability of the DDM is a motivating factor in our decision to use it as a basis for our study. We note that the DDM is much simpler than the leaky competing accumulator (LCA) Model (Usher and McClelland, 2001), which has been used in prior models of sequential effects (Cho et al., 2002; Jones et al., 2002; Gao et al., 2009). LCA processes involve two or more coupled non-linear and stochastic differential equations. We compare the adapted DDM with the LCA-based Cho et al. (2002), Jones et al. (2002), and Gao et al. (2009) models in Section 3.1, using the data of Experiment 1.

#### 2.3.1 Priming mechanism

As with other sequential effects models (e.g., Cho et al., 2002; Jones et al., 2002; Gao et al., 2009), parameters are updated by a priming mechanism to reflect the stimulus history of repetitions and alternations. In the Cho, Jones, and Gao Models, priming is implemented by small history-based changes to the drift parameter, μ. In contrast, in our adapted DDM we update the initial conditions at trial *n* + 1 by setting

(7) $${\tilde{x}}_{0}(n+1)=\pm k(M(n)-\frac{1}{2})\pm {\tilde{x}}_{\text{offset}},$$

in which *n* is the previous trial number, *k* > 0 is a scaling constant, and *M*(*n*) serves as a dynamic memory of repetitions and updates at the start of each new trial. *M*(*n*) is confined to the interval [0,1], so that *M*(*n*) − 1/2 ranges from −1/2 to 1/2. A symmetry between R and A biases is then enforced: a positive value of *M*(*n*) − 1/2 corresponds to bias toward R trials and a negative *M*(*n*) − 1/2 corresponds to bias toward A trials. Moreover, updates to *M*(*n*) are defined such that an increase in bias toward R trials will correspond to a decrease in bias toward A trials, and vice versa. Without loss of generality, we define our model terms such that the positive direction for ${\tilde{x}}_{\text{offset}}$ always corresponds to the correct response. The normalized drift parameter $\tilde{\mu }$ must then always take a positive value, and the sign of the offset bias ${\tilde{x}}_{\text{offset}}$ and the scaling constant *k* will vary from trial to trial, with positive coefficients selected if the current trial is a repetition of the previous stimulus and negative coefficients if it is an alternation.

The memory function is updated as follows:

(8) $$M\left(n\right)=\Delta M\left(n-1\right)+\left\{\begin{array}{cc} 1-\Delta ,\; & \text{if}\;\text{repetition}\;\text{from}\;n-1\;\text{to}\;n,\\ 0,\; & \text{if}\;\text{alternation}\;\text{from}\;n-1\;\text{to}\;n,\\ \; \end{array}\right.$$

where 0 < Δ < 1. The Δ parameter determines the dependence of behavior on previous trials, with higher values corresponding to the level of influence of trials further back in the sequence and lower values corresponding to dependence on only recent trials. A Δ value of 0.5 corresponds to a memory length of approximately four trials (Δ^4^ = 0.0625), after which history dependence goes below 5%. A single update parameter Δ can then account for responses to both R and A trials. In contrast, the Cho, Jones, and Gao models used a memory function *M*(*n*) but separately tracked R and A trials. Our model is always initialized with no bias, so that *M*(1) = *M*(2) = 1/2, after which *M*(*n*) updates according to the above expression. This mechanism allows for large adjustments to initial conditions to follow the termination of strings of repetitions or alternations. The updating mechanism is similar to updates to biasing terms proposed in previous work (Cho et al., 2002; Gao et al., 2009), where initial conditions and drift rates are updated.

#### 2.3.2 Error-correcting mechanism

We also employ error-correction threshold modulation. Threshold modulation has been studied in the context of several sequential choice tasks (Bogacz et al., 2006; Simen et al., 2006). In particular, models have used variable thresholds in describing optimal behavior, as well as to account for variability in reaction time. Increased caution is attributed to a higher threshold, which is understood to follow error commission. However, prior models of sequential effects have not included threshold modulation.

In the adapted DDM, the thresholds are adjusted after every trial and constrained to remain symmetric at $\pm \tilde{z}$. After a correct trial, $\tilde{z}$ is reduced by ${\tilde{z}}_{\text{down}}>0,$ and after an error trial, increased by ${\tilde{z}}_{\text{up}}>0:$

(9) $$\tilde{z}\left(n\right)=\tilde{z}\left(n-1\right)+\left\{\begin{array}{cc} -{\tilde{z}}_{\text{down}},\; & \text{if}\;\text{correct}\;\text{at}\;n-1,\\ {\tilde{z}}_{\text{up}}\; & \text{if}\;\text{error}\;\text{at}\;n-1.\\ \; \end{array}\right.$$

The range of $\tilde{z}\left(n\right)$ is constrained so that the thresholds always have a magnitude greater than or equal to the magnitude of the initial conditions, i.e., such that $\tilde{z}(n)\geq \frac{k}{2}+{\tilde{x}}_{\text{offset}};$ $\tilde{z}\left(n\right)$ is also constrained so that $\tilde{z}(n)\leq {\tilde{z}}_{\text{max}}.$ The thresholds are initialized conservatively such that $\tilde{z}(1)={\tilde{z}}_{\text{max}}.$ If an update causes $\tilde{z}\left(n\right)$ to fall outside its bounds, $\tilde{z}\left(n\right)$ is then set to the value of the nearest bound until the next trial.

Sequential, error-correcting variations in the evidence thresholds $\tilde{z}\left(n\right)$ can produce significant differences between reaction times for correct and error trials. Trials with lower thresholds have higher ERs and faster RTs; thus, on average, error trials are faster and correct trials slower. This effect is modulated by adjustments to the initial condition ${\tilde{x}}_{0}$ which result in faster correct or incorrect responses by biasing the system asymmetrically toward one of the choices. The memory function and initial condition and threshold updates add six parameters to the model: *k*, ${\tilde{x}}_{\text{offset}}$ Δ, ${\tilde{z}}_{\text{down}}$ ${\tilde{z}}_{\text{up}}$ and ${\tilde{z}}_{\text{max,}}$ in addition to $\tilde{\mu }$ and *T*_nd_, for a total of eight parameters.

### 2.4 Model simulation and data fitting procedure

Fitted model parameters were used to validate the adapted DDM against data from the two experiments. Separate analysis and fitting was conducted for Experiments 1 and 2. In each case, the data were sorted by sequence, RT, and ER. Model behavior was computed for each parameter set and then sorted similarly. The model was run using the same stimulus sequences that each participant had encountered. Parameters were selected by attempting to minimize the sum of squares error between model prediction and participant data,

(10) $$\text{Err}=\overset{N}{\underset{i}{\sum }}\;{\left(r_{i,\text{model}}-r_{i,\text{data}}\right)}^{2},$$

in which the elements *r_i_* include unweighted overall mean RTs for each of the four possible second-order sequences for repeating (R) and alternating (A) stimuli. We considered RR, AR, RA, and AA sequences for correct trials, for error trials, and for trials overall, mean ERs for these sequences, as well as mean RTs before error trials, on error trials, and after error trials. For Experiment 1, *r* had *N* = 19 elements. For Experiment 2, *r* had *N* = 3 × 19 = 57 elements, because data was included for each of the 3 values of *P*_A_. Time was considered in units of seconds and ERs in decimal fractions of trials, so that range of parameters for elements of *r* were comparable.

The search for parameters was conducted using a Trust-Region-Reflective Optimization (TRRO) algorithm (Coleman and Li, 1994, 1996). The function lsqnonlin in Matlab was used with default options to search and select parameters that minimize equation (10). For each parameter set and experimental condition, the model ran at least 5 times through the stimulus sequence that each participant had encountered in a given block of trials. (Thus, if a participant were to see left, then left, then right stimuli, the model was presented with those same stimuli in sequence left-left-right, along with the stimuli preceding and following them, and these entire sequences would be repeated for the model subject at least 5 times.) For each trial the probability of error was computed from equation (3) and from this number the correctness or error of that trial was decided by biased coin flip. The expected correct or error RT for the trial was then obtained from equation (5) or equation (6), and parameter updates were implemented according to equations (7–9). The individual trial results were then sorted and averaged in the same manner as the experimental data, model predictions were inserted into equation (10), and model parameters were updated by the TRRO algorithm. This was repeated until the lsqnonlin convergence criterion was met. To produce the model data plots in Section 3, each model with its best fit parameters was rerun 10 times and the resulting RTs and ERs computed by averaging over these runs.

Use of the analytical expressions of equations (3–6) for expected ERs and RTs substantially speeds up the fitting process, since direct numerical simulations of equation (1) are avoided. The final parameter selections are listed in Table 1, and the results and implications of the fitting process are considered in the results and discussion sections of this paper.

**Table 1:** **DDM parameterization**.

	$\tilde{\mu }$	*T*_nd_	*K*	${\tilde{x}}_{\text{0,offset}}$	Δ	${\tilde{z}}_{\text{down}}$	${\tilde{z}}_{\text{up}}$	${\tilde{z}}_{\text{max}}$
Experiment 1	38.1747	0.2626	0.0943	0.0051	0.6860	0.0058	0.0348	0.2857
Experiment 2	19.3312	0.3359	0.1181	0.0034	0.6882	0.0034	0.1635	0.2062

A study of the differences between the two tasks can lend some insight into the different fit parameterizations for each of the experiments. We note that the choice tasks presented in Experiment 2 are more challenging than those of Experiment 1, in which stimuli were highly discernable. The difference in signal to noise ratios ($\tilde{\mu }$) in the fits to the two experiments is therefore to be expected. In addition, more difficult tasks generally incur more conservative or cautious behavior in subjects, even when it is not in the subjects’ best interests (Bogacz et al., 2006). Increased caution (and consequently higher thresholds in DDM fits) have been shown to correspond to more difficult tasks (e.g., Ratcliff et al., 2000, 2001, 2004). Thus, after correct responses in Experiment 2, model threshold adjustments ${\tilde{z}}_{\text{down}}$ are small, whereas in Experiment 1 they are larger, and corrections after errors ${\tilde{z}}_{\text{up}}$ are smaller in Experiment 1 than in Experiment 2. Our Δ values are consistent with studies showing stimulus history dependence of up to 4 trials back (e.g., Soetens et al., 1984). The remaining parameters are relatively closer in magnitude for both experiments.

## 3 Results

In order to better understand the relationship between sequential effects and error effects, data from the two experiments were sorted by stimulus sequence and response correctness and compared with model predictions. We first note several trends from this analysis in the Experiment 1 data. We then analyze data from Experiment 2, and we consider how error and sequential effects are influenced by the relative frequencies of repetition (R) and alternation (A) trials. At the same time, we validate our model fits by comparing them with the data from the two experiments.

In our analysis, we refer to RA and AR sequences as *unexpected sequences*, and RR and AA sequences as *expected sequences*. The RT for an RA sequence is the RT corresponding to the A trial, and for an AR sequence, the RT corresponding to the R trial. We call an *R line* one which connects plotted data for RR and AR, and an *A line* one which connects plotted data for RA and AA. We consider only the two most recent trials in each sequence in our calculations, as the effects of errors are known to have a limited duration (Rabbitt, 1966); moreover, for the strongly biased stimuli (*P*_A_ = 10, 90%) of Experiment 2, longer sequences of A’s, respectively, R’s, occur too rarely to yield sufficient data. The degrees of freedom for the *F*-tests were Greenhouse-Geisser adjusted for all reported main effects and interactions in which there were significant violations of sphericity.

### 3.1 Experiment 1: Error dynamics in unbiased tasks

**Figure 2:**
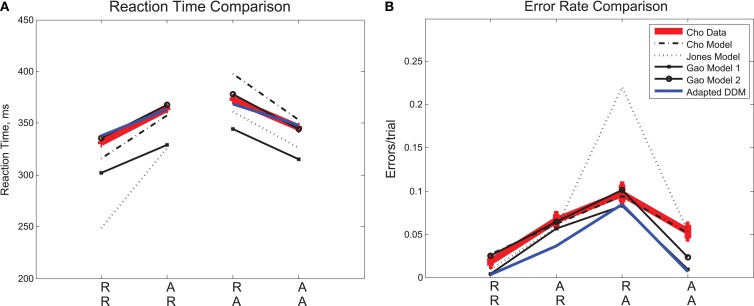
**Mean (A) RTs and (B) ERs for the data in Experiment 1 (Cho Data), the three published fits to the data (Cho, Jones, Gao), and the fit presented for this data in the adapted DDM of the present study**. In the diagram, an RR sequence refers to the RT on the second repetition of a stimulus (e.g., left, left, *left*) and an AR sequence refers to the RT on the first repetition of a stimulus (e.g., left, right, *right*), etc. The adapted DDM provides the best fit to RTs, but underestimates errors, especially for AA sequences.

We consider sequential effects and error effects in Experiment 1 data (referred to as Cho Data), in which R and A trials were equally likely, and as has been customary, we initially average over all responses, correct and incorrect. We first discuss overall sequential effects in RT and ER, as shown in Figure 2. As expected, we find the smallest mean RT and ER for expected trials (RR, AA), and the largest mean RT and ER for unexpected trials (AR, RA). The effects of sequence on RT [*F*(3,15) = 14.81, *p* < 0.001, η^2^ = 0.13] and ER [*F*(3,15) = 8.80, *p* < 0.01, η^2^ = 0.25] were significant in two one-way, within-groups ANOVAs. We consider also three published, generative models of the data in Experiment 1, which we refer to as the Cho et al. (2002), Jones et al. (2002), and Gao et al. (2009) Models, respectively. These models were designed to account for these basic sequential effects, and we note that they, as well as the adapted drift diffusion model (DDM) described in Section 2.3, account for trends in mean RTs and ERs.

**Figure 3:**
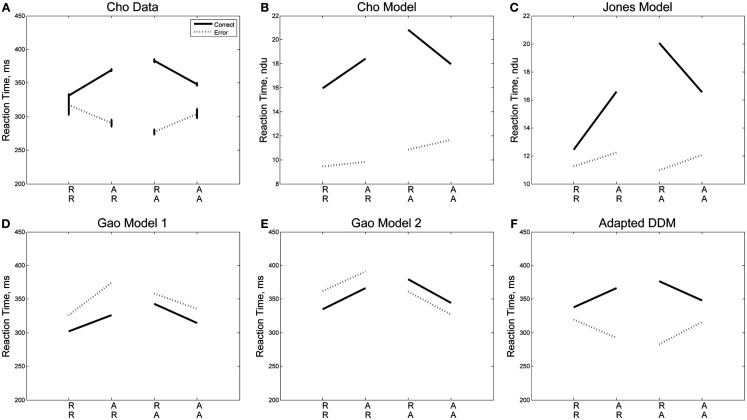
**Sequential effects in Experiment 1: (A) data, (B) results of the Cho Model (Cho et al., 2002), (C) results of the Jones Model (Jones et al., 2002), (D,E) results of the Gao Models (Gao et al., 2009), and (F) results of the adapted DDM**. The Cho and Jones models predict a dimensionless reaction time, which we give here in non-dimensional units **(B,C)**. The adapted DDM captures the slopes of the R and A curves for error and correct trials. In these figures, the correctness or lack thereof of a given trial corresponds only to that trial itself, so a left-right-left sequence is tabulated as correct for the final left stimulus if and only if the final trial were identified correctly as a left stimulus.

We next consider the data separated into correct and error trials, shown in Figure 3A as solid and dotted lines, respectively. Splitting the data in this way reveals a separation in mean RT for correct and error responses that is greatest for unexpected trials (AR, RA) and least for expected ones (RR, AA). For unexpected trials, error responses are fast, and correct responses are slow. A two-way within-groups ANOVA shows the effect of response correctness is significant [*F*(1,5) = 113.93, *p* < 0.001, η^2^ = 0.35)], along with the interaction of response correctness and expectedness of a stimulus [*F*(1,5) = 16.51, *p* < 0.01, η^2^ = 0.19]. We note a slight asymmetry in the responses such that RTs for error and correct trials are closer for the R lines than for the A lines. Figures 3B,C illustrate the results of the Cho and Jones Models, respectively, and Figures 3D,E those of the two versions of the Gao Model. While all four of these models capture the trends in RT for correct trials, none of them predicts the qualitative patterns or quantitative results for error trials. Since the ERs are generally low, RTs averaged over both correct and error trials are close to the mean RTs for the correct trials alone, and this failure of the models becomes apparent only when error trials are considered separately (cf. Figure 2, which displays fairly good fits, and see Cho et al., 2002; Jones et al., 2002; Gao et al., 2009). This analysis also reveals that the errors, while fast on average, are not uniformly so, being significantly faster for unexpected sequences (AR, RA). Moreover, as shown in Figure 3F, the adapted DDM accounts for all the RT data.

**Figure 4:**
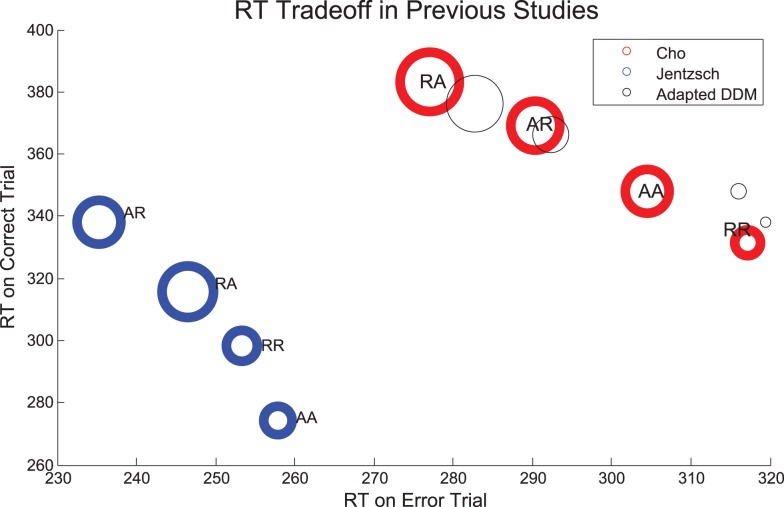
**Sequential RT tradeoff for unbiased tasks: a slower RT for correct trials corresponds to a faster RT for error trials for the sequences RR, AR, RA, and AA**. The RT tradeoff for Experiment 1 is shown in red. Also shown, in blue: the RT tradeoff from a prior study by Jentzsch and Sommer (2002). Adapted DDM fits to Experiment 1 data are shown in black. The areas of the circles are proportional to the ERs.

Strikingly, we note that when plotted against each other as in Figure 4, RTs for correct and error trials for the sequences RR, AR, RA, and AA display a monotonic and nearly linear relationship, which we call the *sequential RT tradeoff*. As we shall see, such a tradeoff also holds for Experiment 2. In Figure 4 we show the data from Experiment 1 (*R*^2^ = 0.995, *p* < 0.01) and the adapted DDM, and from a separate study by Jentzsch and Sommer, 2002; *R*^2^ = 0.96, *p* < 0.05). The area of the circles are proportional to the ERs for the given sequences. We note that the smallest ERs correspond to sequences with relatively fast correct responses and slow errors, while the high ERs occur with relatively fast errors and slow correct responses. While the overall ordering of the sequences (RR, AR, RA, AA) in the tradeoff differs between the two experimental studies, in both cases the points corresponding to unexpected trials (AR, RA) lie at the upper left, and those corresponding to expected trials (RR, AA) lie at the lower right.

The ordering of the tradeoffs is influenced by the nature of the task. However, in each task we see that an increase in time to respond correctly (or a bias toward the correct response) is correlated with a decrease in time to respond in error, and vice versa. Our proposed biasing mechanism achieves a similar effect.

**Figure 5:**
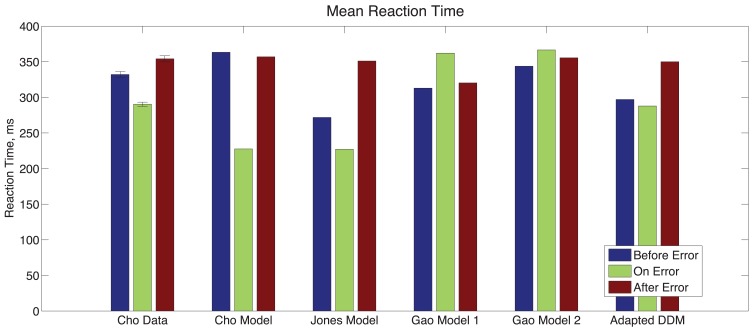
**Post-error slowing in Experiment 1 data and in the models of Cho et al. (2002), Jones et al. (2002), Gao (first model; Gao et al., 2009), and the adapted DDM of the present study**. In the Experiment 1 data, the mean RT for a trial immediately following the error trial is slower than that for the trial before the error, and the mean RT for the error trial itself is fast. The Cho and Gao models fail to account for both trends. The Jones Model accounts for the proper trends but overestimates the magnitude of the post-error slowing. The adapted DDM accounts for both trends but underestimates post-error RTs.

Finally, we consider the RTs before, during, and after an error in Experiment 1, as shown in Figure 5. Mean RTs for trials immediately following an error are longer than both those for the error trial itself and for the trial immediately before the error. A one-way within-groups ANOVA confirms that this effect on RT is significant [*F*(2,10) = 16.37, *p* < 0.001, η^2^ = 0.48]. We again compare the behavior with the adapted DDM and the three previous models. In the Cho Model, the RT after an error is slower than the RT on the error trial but faster than the trial immediately prior to the error. The Jones Model maintains the trends in the data but parameter values are skewed so that the range of RTs is larger. In the two Gao Models, mean RTs for trials immediately preceding and following an error are faster than those on the error trial itself: opposite to the data. The adapted DDM provides the best fit, with the RTs for error trials and post-error trials closely matching the data, although it underestimates RTs on the pre-error trial.

We compare the adapted DDM with the other models using the Akaike Information Criterion (AIC; Akaike, 1974; Stone, 1979), corrected AIC (AIC_c_; Hurvich and Tsai, 1989; Burnham and Anderson, 2002), and Bayesian Information Criterion (BIC; Akaike, 1980; Smith and Spiegelhalter, 1980), which provide model fit comparisons that account for the number of parameters included in each model. Scores for the different model fits are shown in Table 2. The adapted DDM receives the best overall and relative scores on all three metrics, confirming the fit qualities shown in Figures 2–5. AIC_c_ values cannot be computed for the Gao model, because the number of means being compared is too close to the number of parameters used in the model itself.

**Table 2:** **Model performance comparison**.

Model	Total parameters	*R*^2^	AIC	AIC_c_	BIC
Adapted DDM	8	0.996	84.7	115.1	108.2
Cho	13	0.936	138.2	237.0	176.5
Gao 1	18	0.915	140.4	–	193.4
Gao 2	18	0.951	137.0	–	190.0
Jones	16	0.943	146.9	450.9	194.1

### 3.2 Experiment 2: Influence of stimulus alternation frequency

**Figure 6:**
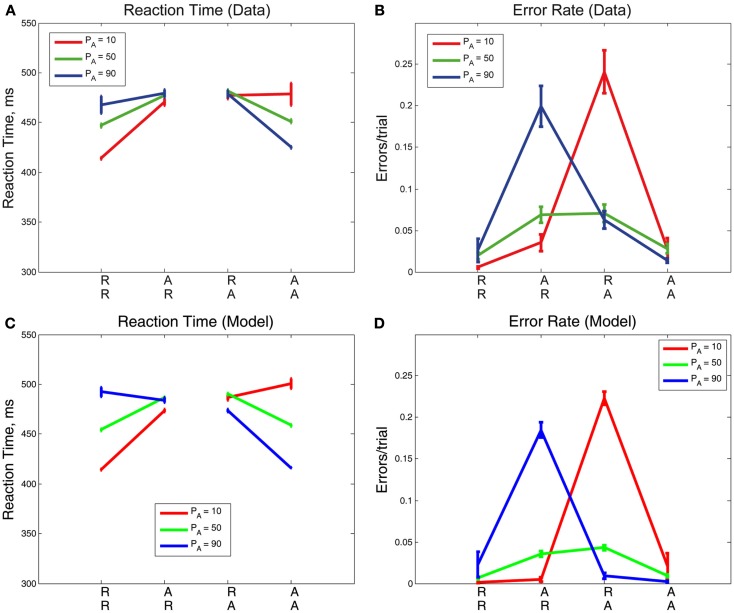
**Mean (A) RTs and (B) ERs for the three values of *P*_A_ in Experiment 2, averaged over correct and error trials**. The influence of *P*_A_ is most apparent in the mean RT plot on expected trials (RR, AA) and in the mean ER on unexpected trials (AR, RA). Model fits for **(C)** RTs and **(D)** ERs re-create behavioral trends in RTs and ERs but overestimate RTs for expected trials (RR, AA). The error bars in plots **(A,B)** represent the standard error of the mean, and in **(C,D)** the average value of standard error of the mean over 10 simulation runs (see Section 2.4 for details).

We now consider the role that alternation frequency plays in sequential and error effects. We first address overall trends in RT and ER, as shown in Figures 6A,B, respectively, following the convention in the sequential effects literature (e.g., Soetens et al., 1985; Jentzsch and Sommer, 2001; Cho et al., 2002). Trends for the *P*_A_ = 50% blocks match trends from Experiment 1 with longer RTs and higher ERs for unexpected trials, and shorter RTs and lower ERs for expected trials. Trends for the *P*_A_ = 10% blocks and *P*_A_ = 90% blocks are clearly distinguishable from the trends for *P*_A_ = 50% blocks, notably in the magnitudes of the slopes of R and A lines. Further, there is an approximate symmetry between the *P*_A_ = 10% case and the *P*_A_ = 90% case.

Sequential effects in mean RTs are clearly influenced by the probability of alternations, with respect to both overall mean RTs and ERs (Figures 6A,B). Mean RTs for unexpected sequences (AR, RA) remain similar for all *P*_A_ conditions but there are significant differences in mean RTs for expected sequences (RR, AA). For the highest *P*_A_, RT is faster on AA trials than the corresponding sequence RTs for lower *P*_A_s, and for the lowest *P*_A_, the RT is faster on RR trials than the corresponding sequence RTs for higher *P*_A_s. As expected, we find that the effects of sequence [*F*(3,42) = 50.62, *p* < 0.001, η^2^ = 0.26] and its interaction with *P*_A_ [*F*(3.36, 47.04) = 43.09, *p* < 0.001, η^2^ = 0.26] on RT are both significant. Error rates are greatest for AR trials at the highest *P*_A_ and RA trials at the lowest *P*_A_. The effects of sequence [*F*(1.95, 27.30) = 20.86, *p* < 0.001, η^2^ = 0.31] and its interaction with *P*_A_ [*F*(2.46, 34.44) = 20.19, *p* < 0.001, η^2^ = 0.32] on ER are also both significant. The adapted DDM reproduces the qualitative patterns in the data, but overestimates RTs for expected sequences when their probabilities are low (RR, with *P*_A_ = 90%; AA, with *P*_A_ = 10%), and underestimates ERs for unexpected sequences (AR, RA): Figures 6C,D.

We also found that the overall sequential effects are influenced by the probability of alternations. The relationship between the time to respond to sequences ending in R versus A on the final sequence is known to indicate relative preference for R or A trials (Audley, 1973). Prior work has shown that preference for A trials varies with RSI, but the role of the likelihood of A trials in determining the relative preference for A has not been studied.

**Figure 7:**
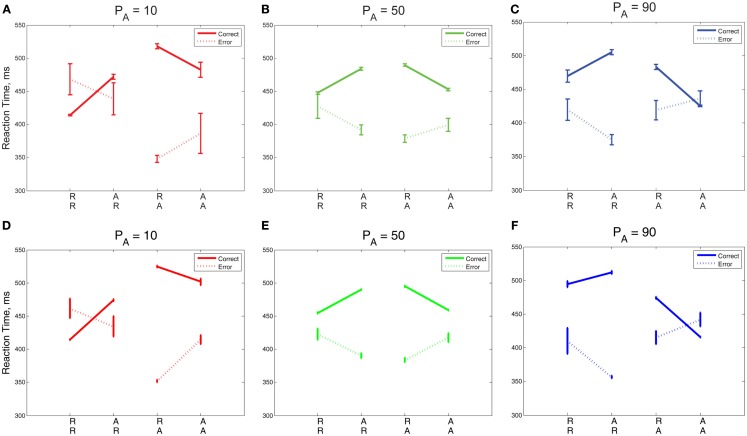
**(A–C)** RT data for error and correct trials in Experiment 2 compared with the adapted DDM **(D–F)**. The slopes of the R and A lines are reversed for correct and incorrect responses. Error trials incur faster responses on unexpected trials (RA, AR) than on expected trials (RR, AA); this trend is reversed for correct responses. For low *P*_A_, the R lines overlap, and for high *P*_A_, the A lines overlap, resulting in an approximate reflectional symmetry between data for the high and low *P*_A_ blocks, so that sometimes the mean time for an error trial is slower than for a correct trial. The error bars in plots **(A–C)** represent the standard error of the mean, and in **(D–F)** the average value of standard error of the mean over 10 simulation runs (see Section 2.4 for details).

The green lines corresponding to *P*_A_ = 50% in Figures 7A,C show no preference for R or A: expected sequences (RR, AA) yield faster RTs symmetrically in R and A than unexpected sequences (AR, RA). The red *P*_A_ = 10% blocks show a strong preference for R: the mean RT after an R is faster in the case of RR than it is for AR, whereas the RT after A is similar for both RA and AA. The blue lines corresponding to *P*_A_ = 90% show a strong preference for A: the RT after an A is faster in the case of AA than it is for RA, whereas the RT after an R is similar for both RR and AR. For *P*_A_ = 10%, the model predicts, as in the data, that the repetition RT is faster for RR than it is for AR, but the model predicts a slower alternation RT for AA than for RA, and it shows a symmetric trend for *P*_A_ = 90%. In summary, both data and model exhibit increases in preference for A with increased probability of alternations, showing that relative preferences for R or A trials can be influenced by transition probabilities in addition to task properties such as RSI.

In Figures 7A–C we replot the mean RT data, separated into correct and incorrect responses, thus revealing differing sequential effects for each *P*_A_. A two-way within-groups ANOVA shows that the effects of correctness [*F*(1,14) = 249.64, *p* < 0.001, η^2^ = 0.80], whether or not the trial was expected [*F*(1,14) = 54.70, *p* < 0.001, η^2^ = 0.44], and the interaction of these two factors [*F*(1,14) = 88.38, *p* < 0.001, η^2^ = 0.62] are all significant. For unbiased sequences (*P*_A_ = 50%), sequential effects are again similar to those for correct and error trials in Experiment 1 (Figure 7B, cf. Figure 3A). For both low and high *P*_A_ blocks, the orientations of the R and A lines are maintained, with correct R lines sloping upwards from RR to AR and correct A lines sloping down from RA to AA. For *P*_A_ = 50%, the slopes of the R and A lines for incorrect responses are nearly opposite the slopes of the R and A lines for correct responses. For *P*_A_ = 10% blocks, the R lines cross and the A lines are further apart than in the *P*_A_ = 50% blocks. For *P*_A_ = 90% blocks, we see a mirrored trend, in which the A lines cross and the R lines are further apart than in the *P*_A_ = 50% block. We also note a striking asymmetry for the biased stimuli: for *P*_A_ = 10% R lines are, on average, closer together than A lines, and for *P*_A_ = 90% this relationship is mirrored, so that A lines are closer than R lines. However, the mirroring is not perfect: the degree of overlap in R lines is greater for *P*_A_ = 10% than the corresponding overlap in A lines for *P*_A_ = 90%.

The trends in correct and error trial RTs, including the crossover of the R and A lines, are generally captured by the adapted DDM, as shown in Figures 7D–F. However, the steepness of slope of the R (respectively, A) lines are underestimated for correct trials for *P*_A_ = 90% (10%), due to overestimation of the RR (AA) RTs.

**Figure 8:**
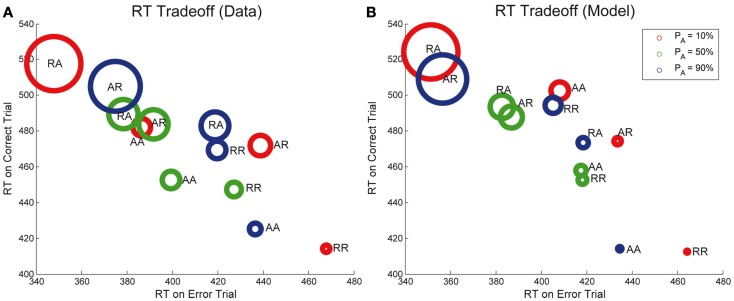
**Sequential RT tradeoff for Experiment 2**. Mean RTs for correct trials are strongly correlated with mean RTs for error trials for each of the sequences RR, AR, RA, AA, for each value of *P*_A_ for both **(A)** data and **(B)** adapted DDM.

Next, we note that the sequential RT tradeoff between correct and error responses is also observed in Experiment 2, as shown in Figure 8A. As in Figure 4, the areas of the circles are proportional to the corresponding ERs. The relationship between RTs for correct and error trials for each of the sequences RR, AR, RA, and AA is monotone (and nearly linear) for all points shown in the figure (*R*^2^ = 0.75, *p* < 0.001), and this correlation is also captured by our model (*R*^2^ = 0.74, *p* < 0.001). The sequences with the largest ER have relatively fast RTs for errors and relatively slow RTs for correct trials. Note, however, that data for individual *P*_A_^’^s of 10, 50, and 90% is not quite as strongly correlated. Differences in order can be expected because the sequential effects for each probability of alternation are influenced by the probability of alternation.

**Figure 9:**
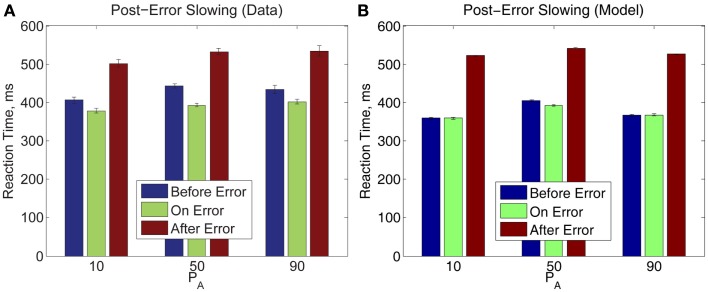
**(A)** Post-error slowing in Experiment 2 data is independent of *P*_A_. **(B)** The model fit also predicts post-error slowing but does not fully account for pre-error speeding. The error bars in plot **(A)** represent the standard error of the mean, and in **(B)** the average value of standard error of the mean over 10 simulation runs (see Section 2.4 for details).

Finally, we note that post-error slowing occurs for all *P*_A_ blocks with the same trend: the error trial itself incurs a slightly faster RT than the trial which precedes it, and the post-error trial incurs an RT significantly slower than RTs for the preceding two trials, as shown in Figure 9. A two-way within-groups ANOVA indicates that the effects of time before, upon, and after an error commission [*F*(1.36, 19.04) = 68.25, *p* < 0.001, η^2^ = 0.57] and on *P*_A_ [*F*(2,28) = 5.83, *p* < 0.01, η^2^ = 0.07] are both significant, but the effect of their interaction is not significant. Thus, in Experiment 2, pre- and post-error RTs share the pattern of RTs in Experiment 1, and this pattern is preserved over all three values of *P*_A_. The bottom panel shows that our model both qualitatively and quantitatively captures the post-error slowing in Experiment 2. However, as in Experiment 1 (Figure 5), the model fails to produce the observed speed-up on the error trial itself.

## 4 Discussion

In this paper, we propose priming and error-correcting mechanisms to account for sequential effects and post-error slowing, respectively. Each mechanism, on its own, is commonplace in models of decision making. Indeed, various priming mechanisms have been previously proposed to account for sequential effects (Cho et al., 2002; Jones et al., 2002; Gao et al., 2009). Post-error slowing is also known to occur and exert a significant influence on RT patterns (Rabbitt, 1966, 1968a; Laming, 1979b). The implementation of post-error slowing is understood to be a simple one: in an accumulator model, the response thresholds can be raised following an error to increase the necessary processing time before a decision is reached (Rabbitt and Rodgers, 1977; Rabbitt and Vyas, 1981; Brewer and Smith, 1984; Jentzsch and Dudschig, 2009). To the best of our knowledge, no prior model of sequential effects has explicitly incorporated such an error-correcting mechanism to also account for post-error slowing. We consider sequential effects for both high and low probabilities of alternations, a consideration unique to this paper: previously, sequential effects for sequences of alternating and repeating stimuli had been studied only for stimuli in which the probabilities of alternations and repetitions were equal.

Our model is informed by previous work: the initial conditions are varied according to a priming function similar to those in other models (Cho et al., 2002; Jones et al., 2002; Gao et al., 2009), and the thresholds are raised after incorrect responses and lowered after correct ones (Simen et al., 2006). Variability in thresholds of drift diffusion processes during a trial can result in fast errors (Ratcliff and Rouder, 1998). Our implementation, however, is unique: we use both priming and error-correcting mechanisms in the same model. In doing so, we can account for many of the observed trends in behavior.

Our adaptation of the pure drift diffusion model has multiple advantages. The pure DDM is analytically simple, and explicit expressions exist for both RT distributions and accuracy, and separate and closed-form expressions for mean RTs can be derived for correct and error responses, as shown in the Appendix. With non-zero initial conditions, the pure DDM can also account for RT distributions for correct and error trials. Moreover, the priming and error-correction mechanisms that we have proposed are conceptually straightforward. With the error-correction mechanism, our model accounts for post-error slowing: the RT for the trial which immediately follows an error trial is not only significantly slower than the error trial but also slower than the RT for the trial immediately preceding the error. We show that when thresholds are systematically adjusted to account for error and correct responses and priming is implemented, sequential patterns in error and correct response trial RTs emerge and are consistent with participant behavior, as shown in Figures 5 and 9.

Indeed, our adapted DDM predicts the characteristic trends in mean RTs for sequences ending in correct or incorrect responses whereas several other models do not. We show experimentally and for the first time that unexpected trials (AR or RA) result in relatively slow correct responses and fast errors, whereas expected trials (RR or AA) result in relatively fast correct responses and slow errors as shown again in Figures 3 and 7. Our model captures aspects of this behavior with the incorporation of post-error adjustments to the model thresholds: priming accounts for the sequential patterns in RT for correct trials, and error-correction accounts for the patterns in RT for the error trials.

The relationship between RTs for correct and error trials is central to our model: biasing the initial conditions toward expected sequences automatically biases them *against* unexpected sequences. Subjects biased against an unexpected stimulus will then respond to it slowly if they are to respond correctly, and rapidly if they are to respond in error. In contrast, in previous work (Cho et al., 2002; Jones et al., 2002; Gao et al., 2009), the biasing was instead applied to sensitivity to stimulus, so that the relationship between RT for error and correct trials was less direct. Moreover, when biasing is coupled with explicit post-error adjustments, further nuances in the relationship between mean time to respond correctly versus in error may be realized.

Significantly, we also identify a sequential RT tradeoff, in which the correlation between the mean RTs for error and correct trials for each of the sequences (RR, AR, RA, AA) is quite strong: a faster RT on an error response corresponds to a slower RT on a correct response. The correlation between mean RTs for correct and error trials is captured by our model, as shown in Figures 4 and 8.

We then show that sequential effects in mean RTs overall, as well as in mean RTs for correct and error trials, are significantly influenced by the probability of alternations. Our data reveals remarkable near-mirror-symmetry between RT patterns for alternations when the probability of alternations is low and repetitions when the probability of alternations is high: incorrect responses are fast and correct responses are significantly slower. Sequential effects in ER also vary with the probability of alternations. Our model captures this near-symmetry in Figures 6 and 7.

Moreover, we have shown, both in our data and in our model, that an increase in the likelihood of alternations corresponds to an increase in relative preference for alternations. This can be inferred from the RT versus sequence plots in Figure 6A. The change in alternation preference with changing likelihood of alternations suggests that choice behavior can be informed and even manipulated by the probabilistic structure of the environment.

The sequential effects in RT and ER for various probabilities of alternation are of particular interest due to their relevance to prior physiological studies. In particular, previous work has shown that the anterior cingulate cortex (ACC) is sensitive to alternations in a sequence of stimuli and identified corresponding neural signals (e.g., Botvinick et al., 2001). Prior models of sequential effects, such as those of Jones et al. (2002) and Gao et al. (2009) have included a “conflict” signal informed by activity in the ACC, and the signal increases in strength with frequent alternations. However, the near-symmetry of behavior at high and low probabilities of alternations in our data suggests a comparable sensitivity to repetitions and alternations, rather than to alternations alone. Indeed, prior work has suggested that the role of the conflict signal in trials with long RSI, such as those considered in this paper, is a minor one (Jones et al., 2002; Jentzsch and Leuthold, 2005) and secondary to that of explicit error correction. Jones et al. (2002) found that the incorporation of a conflict signal in their model resulted in a small but significant improvement in model fit. For short RSI, however, the role of response conflict is more significant (Jentzsch et al., 2007; Jentzsch and Dudschig, 2009). Future work could further clarify the respective roles of response caution (thresholds) and response conflict (ACC) co-varying RSIs and probabilities of alternation.

Additional directions for future work include a consideration of alternative error-correction and priming mechanisms. For example, the magnitude of adjustments made due to our priming mechanism varies from trial to trial, while adjustments from the error-correction mechanism are consistent. Alternate models in which different update schemes are employed are worthy of consideration. Such a study could allow for further model simplification and provide a stronger account of behavior in choice tasks. Moreover, sufficient data should be gathered so that sequential and error effects can be studied and described for individual participants, by fitting RT distributions for different stimulus sequences and individual participants. Finally, a consideration of human behavior in more difficult tasks, such as those with low or variable stimulus discriminability, or tasks in which the probability of alternations varies during blocks of trials, can build upon our work.

In this paper, we have presented a neurally plausible and conceptually straightforward account of sequential effects and post-error slowing by developing a simple repetition-based priming mechanism, coupled with an error-correction mechanism. We implemented these mechanisms within the context of a pure DDM, so the behavior can be described analytically and in closed form. Despite its simplicity, our implementation of the DDM accounts for nuances in behavior which are not found in previous models. In particular, we identified in our data, and our model accounted for, sequential effects for correct and error trials, as well as for trials during blocks with high and low probabilities of alternations. This suggests that an error-correction process, such as a simple adjustment of response thresholds after each trial, plays an instrumental role in sequential patterns in RT. Future work may further clarify the implementation of the error-correction process and its implications for perceptual decision making tasks.

## Conflict of Interest Statement

The authors declare that the research was conducted in the absence of any commercial or financial relationships that could be construed as a potential conflict of interest.

## Appendix

In this section, we derive the mean reaction time for the drift diffusion model (DDM) conditioned on hitting either the upper *z*_u_ or lower −*z*_l_ boundaries, and for a general initial condition *x*_0_ ∈ (−*z*_l_, *z*_u_).

Suppose that *x*(*t*) is the position of a Brownian particle at time *t*. The dynamics of the movement of this particle are governed by the drift diffusion equation:

(A1) dx=μdt+σdw

(A2) $$x(0)=x_{0}$$

in which μ is the deterministic drift of the particle, *x*_0_ is the starting position, and σ*dW* are independent white noise (Weiner) increments of r.m.s strength σ. We assume that the particle is allowed to move until it hits either an upper boundary *x*(*T*) = *z*_u_ or a lower boundary *x*(*T*) = −*z*_l_ where *T* is the hitting time. In this case, the joint densities of the hitting time for boundaries at *z*_u_ and −*z*_l_ are given by

(A3) $$g\left(t,x\left(T\right)=z_{\text{u}}\right)=\frac{\pi \sigma^{2}}{{\left(z_{\text{u}}+z_{\text{l}}\right)}^{2}}e^{\frac{\mu }{\sigma^{2}}\left(z_{\text{u}}-x_{0}\right)}\overset{\infty }{\underset{n=1}{\sum }}\;ne^{-\alpha_{n}t}\text{sin}\left(\frac{n\pi \left(z_{\text{u}}-x_{0}\right)}{z_{\text{u}}+z_{\text{l}}}\right),\;\;\;t\geq 0,$$

(A4) $$g\left(t,x\left(T\right)=-z_{\text{l}}\right)=\frac{\pi \sigma^{2}}{{\left(z_{\text{u}}+z_{\text{l}}\right)}^{2}}e^{-\frac{\mu }{\sigma^{2}}\left(z_{\text{l}}+x_{0}\right)}\overset{\infty }{\underset{n=1}{\sum }}\;ne^{-\alpha_{n}t}\text{sin}\left(\frac{n\pi \left(z_{\text{l}}+x_{0}\right)}{z_{\text{u}}+z_{\text{l}}}\right),\;\;\;t\geq 0,$$

where $\frac{1}{2}\left[\frac{\mu^{2}}{\sigma^{2}}+{\left(\frac{n\pi \sigma }{\left(z_{u}+z_{1}\right)}\right)}^{2}\right]$ (cf. Feller, 1968; Ratcliff, 1978; Ratcliff and Smith, 2004).

To obtain the conditional densities, one must divide the above equations by the probability of hitting that particular boundary, i.e., g(t|x(T) = *z*_u_) = g(t,x(T) = *z*_u_)/P[x(T) = *z*_u_].) These probabilities are (Feller, 1968)

(A5) $$P\left[x\left(T\right)=z_{\text{u}}\right]=\frac{e^{-2\frac{\mu x_{0}}{\sigma^{2}}}-e^{-2\frac{\mu z_{1}}{\sigma^{2}}}}{e^{-2\frac{\mu z_{\text{u}}}{\sigma^{2}}}-e^{2\frac{\mu z_{1}}{\sigma^{2}}}},$$

(A6) $$P\left[x\left(T\right)=-z_{\text{1}}\right]=\frac{e^{-2\frac{\mu z_{\text{u}}}{\sigma^{2}}}-e^{-2\frac{\mu x_{0}}{\sigma^{2}}}}{e^{-2\frac{\mu z_{\text{u}}}{\sigma^{2}}}-e^{2\frac{\mu z_{1}}{\sigma^{2}}}}.$$

Thus, the mean reaction time conditioned on hitting the upper boundary is given by

(A7) $$\begin{array}{c} {\left\langle T\right\rangle }_{|z_{\text{u}}}=\int_{0}^{\infty }\;tg\left(t|x\left(T\right)=z_{\text{u}}\right)dt\\ =\frac{1}{P\left[x\left(T\right)=z_{\text{u}}\right]}\frac{\pi \sigma^{2}}{{\left(z_{\text{u}}+z_{\text{l}}\right)}^{2}}e^{\frac{\mu }{\sigma^{2}}\left(z_{\text{u}}-x_{0}\right)}\overset{\infty }{\underset{n=1}{\sum }}\;\frac{n\text{sin}\left(\frac{n\pi \left(z_{\text{u}}-x_{0}\right)}{z_{\text{u}}+z_{\text{l}}}\right)}{\alpha_{n}^{2}}. \end{array}$$

Fortunately, a closed-form expression exists for the sum of the infinite series (Prudnikov et al., 1986; Tuerlinckx, 2004):

(A8) $$\sum_{n=1}^{\infty }\frac{n\sin(ny)}{{\left(C^{2}+D^{2}n^{2}\right)}^{2}}=\frac{1}{D^{2}}\left[\frac{\pi y}{4\frac{C}{D}}\frac{\cosh((\pi -y)\frac{C}{D})}{\sinh(\pi \frac{C}{D})}-\frac{\pi^{2}}{4\frac{C}{D}}\frac{\sinh(y\frac{C}{D})}{\sinh^{2}(\pi \frac{C}{D})}\right]$$

We set $C^{2}=\mu^{2}∕2\sigma^{2},$ $D^{2}={\left(\pi \sigma \right)}^{2}∕2{\left(z_{\text{u}}+z_{\text{l}}\right)}^{2},$ and *y* = (*z*_u_ − *x*_0_). After some algebra, we arrive at a closed form for the mean decision time conditioned on hitting the upper boundary:

(A9) $${\langle T\rangle }_{|z_{\text{u}}}=\frac{1}{P\left[x\left(T\right)=z_{\text{u}}\right]}\frac{1}{\mu }e^{\frac{\mu \left(z_{\text{u}}-x_{0}\right)}{\sigma^{2}}}\left[\frac{\left(z_{\text{u}}-x_{0}\right)\cosh\left(\frac{\mu \left(z_{1}+x_{0}\right)}{\sigma^{2}}\right)\sinh\left(\frac{\mu \left(z_{\text{u}}+z_{1}\right)}{\sigma^{2}}\right)-\left(z_{\text{u}}+z_{1}\right)\sinh\left(\frac{\mu \left(z_{\text{u}}+x_{0}\right)}{\sigma^{2}}\right)}{\sinh^{2}\left(\frac{\mu \left(z_{\text{u}}+z_{1}\right)}{\sigma^{2}}\right)}\right].$$

In a similar fashion we obtain the mean decision time conditioned on hitting the lower boundary:

(A10) $${\langle T\rangle }_{|-z_{\text{u}}}=\frac{1}{P\left[x\left(T\right)=z_{\text{u}}\right]}\frac{1}{\mu }e^{\frac{-\mu \left(z_{\text{1}}-x_{0}\right)}{\sigma^{2}}}\left[\frac{\left(z_{\text{1}}-x_{0}\right)\cosh\left(\frac{\mu \left(z_{\text{u}}+x_{0}\right)}{\sigma^{2}}\right)\sinh\left(\frac{\mu \left(z_{\text{u}}+z_{1}\right)}{\sigma^{2}}\right)-\left(z_{\text{u}}+z_{1}\right)\sinh\left(\frac{\mu \left(z_{1}+x_{0}\right)}{\sigma^{2}}\right)}{\sinh^{2}\left(\frac{\mu \left(z_{\text{u}}+z_{1}\right)}{\sigma^{2}}\right)}\right].$$
